# Protease and Hemicellulase Assisted Extraction of Dietary Fiber from Wastes of *Cynara cardunculus*

**DOI:** 10.3390/ijms16036057

**Published:** 2015-03-16

**Authors:** Cinthia Santo Domingo, Marcelo Soria, Ana M. Rojas, Eliana N. Fissore, Lía N. Gerschenson

**Affiliations:** 1 Industry Department, School of Natural and Exact Sciences, Buenos Aires University (UBA), Ciudad Universitaria, 1428 Ciudad Autónoma de Buenos Aires, Argentina; E-Mails: cinthiastd@di.fcen.uba.ar (C.S.D.); arojas@di.fcen.uba.ar (A.M.R.); eliana@di.fcen.uba.ar (E.N.F.); 2 Fellow of the National Research Council of Argentina (CONICET), 1033 Ciudad Autónoma de Buenos Aires, Argentina; 3 Instituto de Investigaciones en Biociencias Agrícolas y Ambientales—INBA (CONICET), School of Agronomy (UBA), 1417 Ciudad Autónoma de Buenos Aires, Argentina; E-Mail: soria@agro.uba.ar; 4 Member of CONICET, 1033 Ciudad Autónoma de Buenos Aires, Argentina

**Keywords:** *Cynara cardunculus*, protease, hemicellulase, biomass to chemicals, inulin, pectin, polyphenols

## Abstract

The action of protease and hemicellulase for the extraction of fractions enriched in soluble fiber from bracts and stems of *Cynara cardunculus* was evaluated. Using a two-factor simplex design comprising protease amounts of 0–200 μL and hemicellulase amounts of 0–200 mg for 5 g of material, we explored the effect of a 5 h enzymatic treatment at 40 °C on the chemical composition and yield of the fractions isolated. The fractions contained inulin and pectin. In general, the protein, inulin, and polyphenol contents and also the yields were higher for fractions obtained from stems. The most marked effects were observed when enzymes were used at higher concentrations, especially for hemicellulase. The inclusion of a pre-heating step increased the yield and the inulin content for fractions isolated from bracts and stems and decreased the protein and polyphenol contents, and the galacturonic acid for bracts. These fractions, in general, contained the polyphenolic compounds monocaffeoylquinic acid, apigenin, and pinoresinol.

## 1. Introduction

The food industry controls the selection process, cleaning, and preparation of raw plant materials for further industrial processing [1], giving rise to remnants that could be reused, since they are a good source of dietary fiber and phytochemicals. According to Goñi and Hervert-Hernández [2], plant food waste contains more dietary fiber than its respective edible portions.

For food companies, vegetable waste processing and its elimination represent important costs, often inaccurately evaluated by the companies themselves. The transformation of these wastes into products with higher added value allows them to reduce the cost of treatment and generates additional profits, contributing to the actual trend of sustainable development and environmental protection.

Globe artichoke (*Cynara cardunculus* var. *scolymus*) is a species of thistle cultivated as a food. The edible portion of the plant consists of the flower buds before the flowers come into bloom. It is a perennial plant native to the Mediterranean region [3]. The ratio of edible fraction/total biomass produced by the plant is very low, being less than 15%–20% of the total plant biomass. This ratio decreases further if the contribution to the total biomass represented by offshoots, removed from the field by common cultural procedures, is also considered [4]. *Cynara cardunculus* is a rich source of bioactive phenolic compounds, and also inulin, other fibers, and minerals [5,6]. For this reason, it is considered a functional food. Vegetable tissues discarded at harvesting or after industrial processing constitute a valuable and renewable source of biopolymers and bioactive compounds. Upgrading of vegetable waste can not only reduce pollution but also add value to the commodity production. The obtention of valuable compounds from wastes contributes to their upgrading. The extraction, fractionation, and isolation of high added-value compounds from food wastes can be performed through different methodologies. According to Galanakis [7], among the numerous methodologies found in the literature, five distinct recovery stages can principally be observed: (1) macroscopic pre-treatment, which aims at the adjustment of the food waste matrix according to the water content, enzymatic activity, and permeability of the bioresource tissues; (2) macro- and micro-molecule separation by alcohol precipitation, the most popular method for the separation of smaller compounds from macromolecules; (3) extraction, which uses different methodologies according to the physicochemical characteristics of the target molecules; (4) isolation or clarification of the target compounds from co-extracted impurities; and (5) product formation (encapsulation or drying), the stage that attends to the target product stabilization.

Inulin is a natural storage carbohydrate comprising a heterogeneous collection of fructose polymers. As human enzymes cannot digest fructans, they reach the colon and serve as a substrate for enterobacterial growth. Functioning as a prebiotic, inulin has been associated with enhancing the gastrointestinal and immune systems. In addition, it has been shown to increase the absorption of calcium and magnesium, influence the formation of blood glucose, and reduce the levels of cholesterol and serum lipids [8,9]. Diets containing fructans selectively stimulate bifidobacteria and make them the predominant species [10], producing an increased fecal content of short-chain fatty acids and a decreased concentration of tumor-promoting substances, such as ammonia [11,12].

Pectins are a type of soluble fiber commonly present in vegetal tissues. Pectic polysaccharides are bioactive macromolecules that consist mostly of polymers rich in galacturonic acid (GalA) and often contain significant amounts of rhamnose, arabinose, and galactose as well as another 13 different monosaccharides. The pectin network must be partially disrupted to enable extraction from the cell wall biopolymer network through the use of calcium-chelating agents, diluted alkali, diluted acid, or cell wall degrading enzymes. It has been suggested that enzymatic digestion is an environmentally friendly method of extraction that contributes to the sustainability of the process involved [13].

Several studies described numerous pharmacological activities associated with the artichoke, such as hepatoprotective, antioxidative, anticarcinogenic, hypocholesterolemic, antibacterial, anti-HIV, bile-expelling, and urinative effects [4,14,15,16]. Moreover, the biological activities of the artichoke have been reported in various studies, mainly the strong antioxidative effects, which are attributed to caffeoylquinic acid derivatives, and flavonoids such as luteolin glycosides [17,18].

Fruits and vegetables are rich in polyphenols with antioxidant activity. These compounds may be classified into different groups as a function of the number of phenol rings that they contain and of the structural elements that bind these rings to one another. They are present in plant tissues associated to polysaccharides. These molecules have demonstrated several biological effects, as tested *in vitro* or *ex vivo*: they can inhibit the proliferation of cancer cells, reduce vascularization, exert antiviral activity, protect neurons against oxidative stress, stimulate vasodilatation, and improve insulin secretion [19,20].

The objective of this study was to investigate the action of protease and hemicellulase as well as of a short heat pre-treatment on the extraction of fractions enriched in soluble fiber from residues (bracts and stems) of artichoke (*Cynara cardunculus*) industrialization, with the purpose of contributing to their utilization through the sustainable obtention of added value products that can be used in the food industry.

## 2. Results and Discussion

Bracts and stems of artichokes purchased in a local market were dried obtaining a powder enriched in cell wall material (CWM). From the latter and by means of ethanol and heating, the alcohol insoluble residue (AIR) was prepared. The yield of AIR was 63 g/100 g CWM for stem and 78 g/100 g CWM for bracts.

The bract AIR was constituted of 86.4 g/100 g of total carbohydrates (cellulose 22.2 g/100 g; galacturonic acid 8.7 g/100 g; inulin 7.9 g/100 g; other non-cellulosic carbohydrates 47.6 g/100 g), 8.5 g/100 g of proteins, and 15.1 g/100 g of lignin. The stem AIR contained 81.3 g/100 g of total carbohydrates (cellulose 18.9 g/100 g; galacturonic acid 11.1 g/100 g; inulin 11.9 g/100 g; other non-cellulosic carbohydrates 39.4 g/100 g), 9.0 g/100 g of proteins, and 14.8 g/100 g of lignin.

### 2.1. Composition and Yield of Isolated Fractions

Stem or bract AIRs were treated with enzymes at a temperature of 40 °C for 5 h and at a pH of 5.2 [21,22,23,24,25,26].

As can be observed in Table 1 and Table 2 in general, the protein, inulin, and polyphenol contents and also the yields were higher for fractions obtained from stems. The galacturonic acid content and, in some cases, the total carbohydrate content were higher for fractions obtained from bracts.

**Table 1:** Composition of fractions isolated from bracts of *Cynara cardunculus.*

Run	Treatment (protease/hemicellulase) ^1^	Total Carbohydrates (g/100 g) ^2^	Proteins (g/100 g)	Galacturonic Acid (g/100 g)	Inulin (g/100 g)	Total Polyphenols (g/100 g GAE) ^3^	Yield (g/100 g AIR)
1	(0/0)	75.0 ± 1.2 a	3.6 ± 0.1 c	38.24 ± 1.15 a	12.26 ± 0.11 a	9.37 ± 0.34 a	3.76
2	(200/0)	75.1 ± 1.5 a	3.8 ± 0.2 c	38.48 ± 3.00 a	12.76 ± 0.40 a	9.60 ± 0.67 a	4.60
3	(0/200)	102.4 ± 8.2 b	2.7 ± 0.1 b	29.76 ± 3.07 b	13.00 ± 1.00 a	7.05 ± 0.72 b	4.17
4	(200/200)	93.0 ± 2.8 b	2.1 ± 0.1 a	42.18 ± 3.87 a	14.70 ± 0.20 b	6.83 ± 0.16 b	2.69
5	(170/100)	76.1 ± 2.4	1.6 ± 0.1	34.67 ± 2.37	12.30 ± 0.30	6.09 ± 0.15	2.82
6	(30/100)	99.5 ± 3.5	2.6 ± 0.4	33.82 ± 4.66	14.30 ± 0.10	8.72 ± 0.97	4.38
7	(100/170)	82.0 ± 1.0	1.9 ± 0.6	34.30 ± 6.89	9.60 ± 0.20	7.98 ± 0.21	3.54
8	(100/30)	75.8 ± 0.6	2.4 ± 0.1	37.64 ± 1.73	12.70 ± 0.00	7.46 ± 0.76	7.75
9	(100/100)	96.4 ± 4.9	2.8 ± 0.0	44.24 ± 4.03	14.20 ± 2.00	8.23 ± 0.61	4.40
10	(100/100)	90.2 ± 1.1	3.2 ± 0.0	33.64 ± 3.68	14.00 ± 0.60	9.35 ± 0.50	3.66
11	(100/100)	76.0 ± 1.5	2.5 ± 0.2	29.94 ± 4.04	11.60 ± 0.20	9.00 ± 0.21	2.89

^1^ The concentration of protease is expressed in μL/5 g AIR. The concentration of hemicellulase is expressed in mg/5 g AIR; ^2^ Calculated in relation to fructose calibration curve; ^3^ GAE: gallic acid equivalents. For runs 1–4, inside each column, different letters indicate significant differences (*p* < 0.05).

**Table 2:** Composition of fractions isolated from stems of *Cynara cardunculus.*

Run	Treatment (protease/hemicellulase) ^1^	Total Carbohydrates (g/100 g) ^2^	Proteins (g/100 g)	Galacturonic Acid (g/100 g)	Inulin (g/100 g)	Total Polyphenols (g/100 g GAE) ^3^	Yield (g/100 g AIR)
1	(0/0)	72.6 ± 3.6 a	4.3 ± 0.4 b	21.33 ± 0.80 a	14.64 ± 2.30 a	11.67 ± 0.44 c	5.09
2	(200/0)	73.8 ± 4.3 a	4.2 ± 0.2 b	18.70 ± 1.52 a	25.61 ± 3.25 b	8.61 ± 0.32 b	8.55
3	(0/200)	87.0 ± 0.4 b	3.1 ± 0.1 a	17.60 ± 2.37 a	11.44 ± 0.06 a	8.16 ± 0.32 ab	6.94
4	(200/200)	82.0 ± 5.0 b	3.9 ± 0.2 ab	18.08 ± 1.75 a	14.85 ± 1.29 a	7.18 ± 0.45 a	6.99
5	(170/100)	77.3 ± 0.8	4.1 ± 0.1	17.73 ± 0.46	15.06 ± 0.21	10.23 ± 0.29	7.43
6	(30/100)	83.9 ± 6.3	3.7 ± 0.5	19.71 ± 1.27	21.27 ± 1.44	9.22 ± 0.06	7.64
7	(100/170)	84.5 ± 5.8	3.9 ± 0.1	14.15 ± 3.26	14.05 ± 1.96	11.16 ± 0.94	10.79
8	(100/30)	70.1 ± 1.7	3.6 ± 0.2	17.64 ± 1.60	19.16 ± 0.23	11.17 ± 0.64	6.49
9	(100/100)	75.6 ± 0.5	3.9 ± 0.1	21.20 ± 0.70	14.87 ± 4.94	11.45 ± 0.76	7.56
10	(100/100)	75.2 ± 1.8	3.5 ± 0.1	20.45 ± 1.63	15.56 ± 0.47	11.54 ± 0.79	5.98
11	(100/100)	78.5 ± 2.1	4.1 ± 0.1	37.09 ± 12.52	17.07 ± 0.60	15.08 ± 0.36	6.61

^1^ The concentration of protease is expressed in μL/5 g AIR. The concentration of hemicellulase is expressed in mg/5 g AIR; ^2^ Calculated in relation to fructose calibration curve; ^3^ GAE: gallic acid equivalents. For runs 1–4, inside each column, different letters indicate significant differences (*p* < 0.05).

With respect to fractions isolated from bracts, it can be observed that: (a) total carbohydrates tended to increase for some treatments when hemicellulase was present at levels of 100–200 mg and (b) proteins and polyphenols tended to decrease when hemicellulase or both enzymes were present.

With respect to fractions isolated from stems, it can be observed that: (a) total carbohydrates tended to increase for some treatments when hemicellulase was present at levels of 100–200 mg; (b) for the higher enzyme additions (runs 2, 3, and 4), polyphenol content tended to decrease; and (c) the yield increased with enzyme presence.

Hemicellulase (**H**) is a group of cell wall degrading enzymes such as xylanases, mannases, and arabinases, which can remove hemicellulose substituents and hence weaken the hemicellulose-cellulose network; the one used in the present research has side-cellulase activity according to providers. Protease (**P**) can degrade extensin, rich in hydroxyproline and present in the cell wall, contributing to cell wall weakening. Consequently, enzymes assayed can alter in a diverse way the cell wall, determining the different characteristics of the fractions obtained.

Fissore *et al.* [16] treated 10 g of artichoke CWM with 250 mg of hemicellulase at pH 5.2 (30 °C, 20 h) and observed that the use of hemicellulase increased the yield and the content of total polyphenols but decreased the content of inulin for both bracts and stems. Furthermore, when hemicellulase was used to isolate pectin from butternut [27], it was observed that the use of this enzyme caused higher yield, higher carbohydrate and protein content, and less galacturonic acid content when compared to a fraction isolated with no enzyme addition.

When the fractions obtained from AIR in the present work are compared to those isolated by Fissore *et al.* [16] from CWM of artichoke, it can be observed that in the former case, fractions contained less protein, less inulin, and more galacturonic acid. Although AIR preparation allowed for concentration of the carbohydrates, eliminating small sugars and other alcohol soluble components, it is possible that protein and inulin have been lost during the heat treatment with ethanol for obtaining AIR, determining the differences observed.

The statistical analysis of the results was performed for bracts and for stems through a regressional analysis and a Pearson correlation.

#### 2.1.1. Regressional Analysis of the Design

The regressional analysis of the results showed that for fractions isolated from bracts, no significant effects of protease and/or hemicellulase concentration were detected in relation to galacturonic acid, inulin, total polyphenol contents, and yield. For carbohydrate content a positive but only slightly significant (*p*: 0.0314) effect of hemicellulase (**H**) was observed with a polynomial model: Y = 85.591 + 9.968 **H**; also, the *R*^2^ of the model was low (0.419). For protein content, a negative and slightly significant effect of hemicellulase (*p*: 0.0412) was observed, with a low *R*^2^ (0.386) and the following polynomial model: Y = 2.65–0.5863 **H**.

For fractions isolated from stems, no significant effects of enzymes were detected for protein and galacturonic acid contents or for the yield. For carbohydrate content a positive and significant effect of hemicellulase (*p* < 0.001; *R*^2^: 0.722) was observed and the polynomial model was Y = 78.2273 + 6.5622 **H**. The inulin content showed a negative and slightly significant effect of hemicellulase with a polynomial model Y = 16.6891–3.5215 **H** (*p*: 0.0382), with a low *R*^2^ (0.396). For polyphenols, a negative effect of protease was observed; the association occurred through the quadratic term (*p*: 0.0476 and *R*^2^: 0.533) and the polynomial model was Y = 11.9468–0.6693P–3.2018 **P**^2^.

#### 2.1.2. Pearson Correlation of the Results

The correlation between data was analyzed through the Pearson product moment coefficient [28] for bracts and stems. The Pearson product moment correlation coefficients (PPMCC) range from −1 (negative dependence) to +1 (positive dependence), and measure the strength of the linear relationship between the variables evaluated. When evaluating each pair of variables, it was observed that for bracts (pairs of data used: 10), carbohydrates-hemicellulase (PPMCC: 0.6766; *p*: 0.0317), and polyphenols-proteins (PPMCC: 0.8584; *p*: 0.0015) were significantly (*p* < 0.05) and positively correlated. For stems (pairs of data used: 9), carbohydrates-hemicellulase (PPMCC: 0.8308; *p*: 0.0055), proteins-protease (PPMCC: 0.8110; *p*: 0.0080) and inulin-hemicellulase (PPMCC: 0.8167; *p*: 0.0072) were significantly correlated (*p* < 0.05) and the coefficient was negative for the last pair. These results show that hemicellulase is linearly related to the content of carbohydrates in the fractions and that protease is related to the content of proteins. We also observed a linear relationship between polyphenol and protein contents, a fact that might be ascribed to protein-polyphenol interactions that were previously reported [29,30,31] and can be responsible for their joint extraction.

### 2.2. Analysis of the Fractions Isolated through Treatments 1–4 and Study of the Effect of a Pre-Heating Step on Their Yield and Composition 

From the data in Table 1 and Table 2, it could be concluded that the most marked effects were observed when enzymes were used at their higher assayed concentrations. Consequently, treatments 1–4 were further investigated including a heating step previous to the enzymatic treatments with the aim of increasing their yield [6,16].

Results obtained are shown in Table 3 and Table 4. Fractions obtained from stems showed, in general, a higher yield and higher protein (when protease is present) and polyphenol (when enzymes are present) content than those obtained from bracts.

**Table 3:** Composition of fractions isolated from bracts of *Cynara cardunculus* after a pre-heating step.

Run BRACTS	Treatment (protease/hemicellulase) ^1^	Total Carbohydrates (g/100 g) ^2^	Proteins (g/100 g)	Galacturonic Acid (g/100 g)	Inulin (g/100 g)	Total Polyphenols (g/100 g GAE) ^3^	Yield (g/100 g AIR)
1	(0/0)	88.0 ± 0.6 a	1.6 ± 0.0 bc	26.13 ± 1.30 a	47.04 ± 0.22 a	2.05 ± 0.12 b	10.92
2	(200/0)	88.3 ± 4.1 a	1.1 ± 0.0 a	19.75 ± 0.70 b	23.28 ± 0.00 b	1.90 ± 0.19 ab	13.25
3	(0/200)	107.0 ± 2.7 b	1.4 ± 0.0 b	19.90 ± 0.20 b	22.44 ± 0.87 b	1.66 ± 0.14 a	12.07
4	(200/200)	90.3 ± 1.5 a	1.7 ± 0.2 c	18.86 ± 0.90 b	30.69 ± 0.18 c	2.48 ± 0.07 c	13.38
ANOVA Table	-	-	-	-	-	-	-
F	-	37.49	24.89	44.31	1863	19.01	-
*p*	-	<0.0001 ***	0.0002 ***	<0.0001 ***	<0.0001 ***	0.0005 ***	-
*R*^2^	-	0.9336	0.9032	0.9432	0.9986	0.8770	-

^1^ The concentration of protease is expressed in μL/5 g AIR. The concentration of hemicellulase is expressed in mg/5 g AIR; ^2^ Calculated in relation to fructose calibration curve; ^3^ GAE: gallic acid equivalents. Inside each column, different letters indicate significant differences (*p* < 0.05). *** *p* < 0.001.

**Table 4:** Composition of fractions isolated from stems of *Cynara cardunculus* after a pre-heating step.

Run STEMS	Treatment (protease/hemicellulase) ^1^	Total Carbohydrates (g/100 g) ^2^	Proteins (g/100 g)	Galacturonic Acid (g/100 g)	Inulin (g/100 g)	Total Polyphenols (g/100 g GAE) ^3^	Yield (g/100 g AIR)
1	(0/0)	84.3 ± 0.9 a	1.7 ± 0.1 a	19.89 ± 0.20 a	39.45 ± 0.50 c	2.03 ± 0.73 a	22.70
2	(200/0)	88.9 ± 0.2 b	2.1 ± 0.1 b	22.81 ± 0.20 b	40.68 ± 0.50 d	3.09 ± 0.14 ab	19.30
3	(0/200)	94.0 ± 0.7 c	1.5 ± 0.1 a	18.25 ± 0.10 c	30.25 ± 0.14 b	2.98 ± 0.07 ab	22.66
4	(200/200)	96.3 ± 1.9 c	2.2 ± 0.1 b	13.73 ± 0.40 d	28.48 ± 0.67 a	3.24 ± 0.38 b	21.47
ANOVA Table	-	-	-	-	-	-	-
F	-	69.76	32.75	691.3	482.4	4.700	-
*p*	-	<0.0001 ***	<0.0001 ***	<0.0001 ***	<0.0001 ***	0.0356 *	-
*R*^2^	-	0.9632	0.9247	0.9962	0.9945	0.6380	-

^1^ The concentration of protease is expressed in μL/5 g AIR. The concentration of hemicellulase is expressed in mg/5 g AIR; ^2^ Calculated in relation to fructose calibration curve; ^3^ GAE: gallic acid equivalents. Inside each column, different letters indicate significant differences (*p* < 0.05). * *p* < 0.05; *** *p* < 0.001.

Data reported in Table 3 and Table 4 were analyzed through ANOVA to evaluate the effect of the different enzymatic treatments involved and Tukey’s *post hoc* test was used to evaluate the specific systems that presented different chemical characteristics with treatment. It can be observed that the use of enzymes produced significant changes in chemical composition (carbohydrates, proteins, galacturonic acid, inulin, polyphenols) of fractions isolated from both tissues.

The same analysis when heat pre-treatment was not applied was performed for data reported in Table 1 and Table 2 (Runs 1–4) and results are reported in Table 5. It can be observed that the use of enzymes produced significant changes in chemical composition (total carbohydrates, proteins, galacturonic acid, inulin, polyphenols) for all the fractions evaluated except for stems, for which galacturonic acid was not significantly (*p* > 0.05) affected by enzymatic treatment.

**Table 5:** One-way ANOVA for the evaluation of the effect of enzymatic treatment for fractions isolated from bracts and stems of *Cynara cardunculus* (Runs 1–4 of Table 1 and Table 2).

	Table	Total Carbohydrates	Proteins	Galacturonic Acid	Inulin	Total Polyphenols
BRACTS	1	-	-	-	-	-
ANOVA Table	-	-	-	-	-	-
F	-	28.30	106.6	9.546	11.11	23.55
*p*	-	0.0001 ***	<0.0001 ***	0.0051 **	0.0032 **	0.0003 ***
*R*^2^	-	0.9139	0.9756	0.7816	0.8064	0.8983
STEMS	2	-	-	-	-	-
ANOVA Table	-	-	-	-	-	-
F	-	9.957	14.20	2.856	26.19	74.97
*p*	-	0.0045 **	0.0014 **	0.1046	0.0002 ***	<0.0001 ***
*R*^2^	-	0.7887	0.8419	0.5172	0.9076	0.9657

** *p* < 0.01; *** *p* < 0.001.

The statistical comparison of results reported in Table 1 and Table 3 and in Table 2 and Table 4 allowed us to conclude that the inclusion of a pre-heating step increased the yield and the inulin content for fractions isolated from bracts and stems and decreased the protein, polyphenol, and galacturonic acid contents for bracts. It is known that below temperatures of ≈50 °C, part of the inulin does not dissolve completely [32]. Most likely, the application of a heat pretreatment at 70 °C helped to increase inulin solubility and its concentration in the extract, from which fractions are insolubilized by means of ethanol. According to Lutz *et al.* [33], the total phenolic content of different cooked vegetables varies depending on the treatment applied. They may decrease up to 50% due to antioxidant breakdown and leaking into the water, may increase due to a higher accessibility, or may remain unchanged. Ruiz-Cano *et al.* [6] studied the effect of thermal treatment on the composition of six artichoke byproducts and observed that thermal treatment had a negative effect on the protein content, probably due to the loss of soluble proteins; also, the total phenolic content varied widely as a function of thermal treatment. Williams *et al.* [34] showed that the levels of phenolics and other bioactive compounds decreased when some vegetables were thermally treated.

Concerning the evaluation of the effect of heat and enzymatic treatment, two-way ANOVA (Table 6) showed that the application of heat did not have the same effect for all enzymatic treatments, revealing the existence of interactions between both treatments except for total carbohydrate content in stems (*p* > 0.05).

**Table 6:** Effect of the application of the enzyme and the heat treatment on the composition of fractions isolated. Significance levels for two-way ANOVA and Tukey’s test.

Significance Levels for Two-Way ANOVA (*p* Values)						
	Source of Variation	Total Carbohydrates	Proteins	Galacturonic Acid	Inulin	Total Polyphenols
**STEMS**	Heat pre-treatment	<0.0001	<0.0001	0.6166	<0.0001	<0.0001
	Enzymatic treatment	<0.0001	<0.0001	<0.0001	<0.0001	<0.0001
	Interaction	0.0885	0.0020	0.0002	<0.0001	<0.0001
**BRACTS**	Heat pre-treatment	0.0002	<0.0001	<0.0001	<0.0001	<0.0001
	Enzymatic treatment	<0.0001	<0.0001	0.0002	<0.0001	<0.0001
	Interaction	0.0041	<0.0001	0.0002	<0.0001	<0.0001

### 2.3. Polyphenol Identification

The study of the phenolics present in the different fractions isolated from artichoke bracts and stems with a pre-heating step was performed using HPLC-DAD-ESI-QTOF-MS operated in negative ionization mode. For the identification, UV spectrum and MS data were used, together with the interpretation of the observed MS spectra in comparison with those spectra found in the literature.

**Figure 1:**
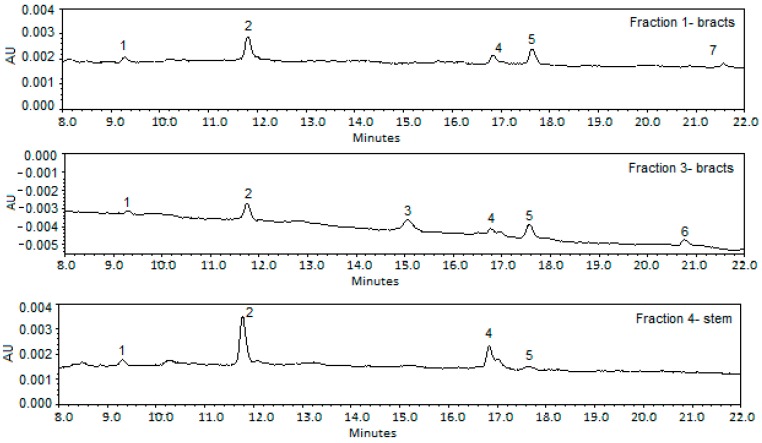
HPLC-DAD chromatograms of fractions isolated from *Cynara cardunculus* after a pre-heating step. Peaks 1: unknown; 2: Monocaffeoylquinic acid; 3: Dicaffeoylquinic acid; 4: Apigenin-7-*O*-glucuronide; 5: Pinoresinol; 6: unknown; 7: unknown.

Figure 1 gives the HPLC-DAD chromatograms of the fractions obtained after a pre-heating step. As can be observed in Table 7 and Table 8, some peaks could not be identified. In all fractions a compound was identified with a mass-to-charge ratio (*m*/*z*) of 353.09, in accordance with the molecular formula C_16_H_17_O_9_ corresponding to monocaffeoylquinic acid. Another compound with an *m*/*z* of 515.12 (molecular formula: C_25_H_23_O_12_), which corresponds to dicaffeoylquinic acid, was observed in Fraction 3, obtained from bracts [35]. These hydroxycinnamic derivatives have been reported previously by Schütz *et al.* [36] who found that the monocaffeoylquinic acids were the major compounds in a total of 13 *Cynara scolymus* commercial preparations (six medicinal, one fresh plant juice, and seven dietary supplements). Pandino *et al.* [37,38,39] also observed that the globe artichoke cultivars “Tondo di Paestum” and “Violetto di Sicilia” were high in caffeoylquinic acids and that, regardless of the cultivar, individual polyphenols were preferentially accumulated in specific parts of the plant, presumably related to their role. In particular, the floral stem was the best source of caffeoylquinic acids, mainly chlorogenic acid (5-*O*-caffeoylquinic acid) and 1,5-di-*O*-caffeoylquinic acid.

With regard to lignans, at 17.6 min three derivatives of pinoresinol not completely separated through HPLC were detected in all fractions with the exception of Fraction 2, obtained from the stems. The detected *m*/*z* of 357.13 corresponds to the molecular formula C_26_H_31_O_11_ and C_28_H_33_O_12_ and the *m*/*z* 151.04 and 136.02 correspond to the molecular formula C_20_H_21_O_6_. According to During *et al.* [40], plant lignans can be absorbed and metabolized in the small intestine, and pinoresinol has a strong anti-inflammatory action on human intestinal Caco-2 cells, possibly in relation to its furofuran structure and/or its intestinal metabolism. 

Concerning flavone derivatives, an apigenin glucuronide was detected in all the fractions through the fragmentation pattern observed in the MS spectrum, which shows a ion at *m*/*z* 269.0462 that corresponds to the molecular formula C_21_H_17_O_11_ [41]. Lombardo *et al.* [42] studied different varieties of globe artichoke and reported that the receptacles are an interesting source of apigenin derivatives, mainly apigenin-7-*O*-glucuronide, and that inner bracts are the richest part in total apigenin among all plant parts analyzed. Navarro Núñez *et al.* [43] noted that the flavonoid apigenin improves the efficacy of aspirin in the inhibition of platelet aggregation.

**Table 7:** Phenolics in fractions ^1^ isolated from bracts of *Cynara cardunculus* after a pre-heating step.

BRACTS	Compound Number	Assigned Identity	R_t_	Formula	M
Fraction 1	1	unknown	9.3	-	-
	2	Monocaffeoylquinic acid	11.8	C_16_H_17_O_9_	353.09
	4	Apigenin-7-*O*-glucuronide	16.8	C_21_H_17_O_11_	445.08
	5	Pinoresinol 4-*O*-β-d-glucoside	17.6	C_26_H_31_O_11_	519.19
		Pinoresinol-acetylhexoside		C_28_H_33_O_12_	561.20
		(+)-Pinoresinol		C_20_H_21_O_6_	357.13
	7	unknown	21.6	-	-
Fraction 2	1	unknown	9.3	-	-
	2	Monocaffeoylquinic acid	11.8	C_16_H_17_O_9_	353.09
	4	Apigenin-7-*O*-glucuronide	16.8	C_21_H_17_O_11_	445.08
	5	Pinoresinol 4-*O*-β-d-glucoside	17.6	C_26_H_31_O_11_	519.19
		Pinoresinol-acetylhexoside		C_28_H_33_O_12_	561.20
		(+)-Pinoresinol		C_20_H_21_O_6_	357.13
Fraction 3	1	unknown	9.3	-	-
	2	Monocaffeoylquinic acid	11.8	C_16_H_17_O_9_	353.09
	3	Dicaffeoylquinic acid	15.0	C_25_H_23_O_12_	515.12
	4	Apigenin-7-*O*-glucuronide	16.8	C_21_H_17_O_11_	445.08
	5	Pinoresinol 4-*O*-β-d-glucoside	17.6	C_26_H_31_O_11_	519.19
		Pinoresinol-acetylhexoside		C_28_H_33_O_12_	561.20
		(+)-Pinoresinol		C_20_H_21_O_6_	357.13
	6	unknown	20.8	-	-
Fraction 4	1	unknown	9.3	-	-
	2	Monocaffeoylquinic acid	11.8	C_16_H_17_O_9_	353.09
	4	*Apigenin-7-italic>O*-glucuronide	16.8	C_21_H_17_O_11_	445.08
	5	Pinoresinol 4-*O*-β-d-glucoside	17.6	C_26_H_31_O_11_	519.19
		Pinoresinol-acetylhexoside		C_28_H_33_O_12_	561.20
		(+)-Pinoresinol		C_20_H_21_O_6_	357.13

^1^ Fractions obtained with a heat pre-heating and with treatment 1 (Fraction 1); treatment 2 (Fraction 2); treatment 3 (Fraction 3); treatment 4 (Fraction 4). For the definition of the different treatments, see Table 1. R_t_: Retention time (min). M: molecular mass (g·mol^−1^).

**Table 8:** Phenolics in fractions ^1^ isolated from stems of *Cynara cardunculus* after a pre-heating step.

STEMS	Compound Number	Assigned Identity	R_t_	Formula	M
Fraction 1	2	Monocaffeoylquinic acid	12.2	C_16_H_17_O_9_	353.09
	4	Apigenin-7-*O*-glucuronide	16.8	C_21_H_17_O_11_	445.08
	5	Pinoresinol 4-*O*-β-d-glucoside	17.5	C_26_H_31_O_11_	519.19
		Pinoresinol-acetylhexoside		C_28_H_33_O_12_	561.20
		(+)-Pinoresinol		C_20_H_21_O_6_	357.13
Fraction 2	2	Monocaffeoylquinic acid	11.8	C_16_H_17_O_9_	353.09
	4	Apigenin-7-*O*-glucuronide	16.8	C_21_H_17_O_11_	445.08
Fraction 3	2	Monocaffeoylquinic acid	11.8	C_16_H_17_O_9_	353.09
	4	Apigenin 7-glucuronide I	16.8	C_21_H_17_O_11_	445.08
	5	Pinoresinol 4-*O*-β-d-glucoside	17.6	C_26_H_31_O_11_	519.19
		Pinoresinol-acetylhexoside		C_28_H_33_O_12_	561.20
		(+)-Pinoresinol		C_20_H_21_O_6_	357.13
Fraction 4	1	unknown	9.3	-	-
	2	Monocaffeoylquinic acid	11.8	C_16_H_17_O_9_	353.09
	4	Apigenin-7-*O*-glucuronide	16.8	C_21_H_17_O_11_	445.08
	5	Pinoresinol 4-*O*-β-d-glucoside	17.6	C_26_H_31_O_11_	519.19
		Pinoresinol-acetylhexoside		C_28_H_33_O_12_	561.20
		(+)-Pinoresinol		C_20_H_21_O_6_	357.13

^1^ Fractions obtained with a heat pre-heating and with treatment 1 (Fraction 1); treatment 2 (Fraction 2); treatment 3 (Fraction 3); treatment 4 (Fraction 4). For the definition of the different treatments, see Table 2. R_t_: Retention time (min). M: Molecular mass (g·mol^−1^).

## 3. Experimental Section

Artichokes were purchased in a local market. The flower head was separated into heart, bracts, and stems. Bracts and stems were cut in small pieces and dried in a convection oven (85 °C, 2.5 h, air rate: 0.5 m/s). The dried product was ground in a domestic grinder (Wemir E909, Buenos Aires, Argentina) to obtain a powder enriched in cell wall material (CWM).

### 3.1. Preparation of the Alcohol-Insoluble Residue (AIR)

According to Martín-Cabrejas *et al.* [44], 100 g of CWM were suspended in 350 mL of 80% (*v*/*v*) ethanol solution, mixed and then boiled for 30 min under stirring. The residue obtained was then boiled once with 350 mL of 80% (*v*/*v*) ethanol solution for 15 min and twice with 250 mL of 80% (*v*/*v*) ethanol solution for 15 min. The insoluble residue was separated and washed with 100 mL of 80% (*v*/*v*) ethanol and finally with 96% (*v*/*v*) ethanol. Between each ethanol treatment, the suspension was filtered and the solvent was discarded. The material was left overnight under a lab hood to eliminate the ethanol and the free ethanol product was freeze-dried (Stokes freeze-drier, Stokes Company, Philadelphia, MA, USA) after freezing at −18 °C. The product was then milled (Wemir E909, Argentina) and stored at −18 °C under a vacuum into heat-sealed Cryovac (polyvinyl chloride-polyvinylidene chloride copolymer) bags until usage.

### 3.2. Isolation of the Fractions 

Since pectin is known to be unstable at pH above 5 when exposed to elevated temperatures [21], it was decided to test enzyme performances at a pH of ≈5, and at a temperature not higher than 50 °C. We also took into account that inulin is stable at pH 5–7 in the temperature range 20–70 °C [22] and its sensitivity to the temperature and acidity of enzyme activities [23,24,25,26]. These facts and the results of preliminary assays determined that the assays were performed at a temperature of 40 °C for 5 h at a pH of 5.2.

An amount of 5.00 g of AIR obtained from artichoke stem or bracts was digested in 500 mL of sodium citrate buffer (0.05 M, pH 5.2) with 0.01% sodium azide (*w*/*v*) and the adequate quantity of enzyme (range of protease concentration: 0–200 μL; range of hemicellulase concentration: 0–200 mg). Hydrolysis was performed with constant stirring (ARE magnetic stirrer, Velp, Usmate, Italy) at 450 RPM for 5 h at 40 °C. The system was filtered through glass fiber filter and two volumes of ethanol 96% (*v*/*v*) were added to the supernatant to precipitate the soluble fiber. After filtration through a glass fiber filter, the solid residue was freeze-dried under the conditions previously described.

A pre-heating step was also assayed, for some selected treatments. This step was performed for 5 min at 70 °C under stirring and was followed by cooling to 40 °C. The system was maintained under constant stirring for 5 h either without or with addition of enzymes.

Enzymes used were: (A) Hemicellulase H2125 (SIGMA, St. Louis, MO, USA): Hemicellulase from *Aspergillus niger* with side cellulase activity. One unit will produce a relative fluidity change of 1 per 5 min, using locust bean gum as substrate at pH 4.5 at 40 °C. This enzyme is named herein as **H**; (B) Protease P1236 (SIGMA, St. Louis, MO, USA): This enzyme is Neutrase^®^ 0.8 L, a protease from *Bacillus amyloliquefaciens* with side endo-proteolytic activity. It has an activity of 0.8 Anson unit/g (Au/g). One Anson unit is defined as the amount of enzyme that digests urea-denatured hemoglobin under specified conditions (25 °C and pH 7.5) at an initial rate such that an amount of trichloroacetic acid (TCA)-soluble product is liberated per minute that gives the same color with the Folin–Ciocalteu Phenol reagent as one milliequivalent of tyrosine. This enzyme is named herein as **P**.

### 3.3. Experimental Design and Statistical Analysis

#### 3.3.1. Experimental Design

In order to find the effect of the factors (protease and/or hemicellulase concentration) on yield and chemical composition of fractions obtained from stem and bracts of *Cynara cardunculus*, a design was used [45]. The levels of enzyme concentrations were arranged as a two-factor simplex design projected from a star and box central composite design (CCD). A two-factor simplex design was chosen over a full factorial design because the former needs 11 treatments, including triplicate central points, in order to cover five different values for each variable over the experimental space. The minimum full factorial design to test curvature would also require 11 treatments, but spanning only three different data values for each variable. The central composite design tests a fraction of all possible combinations of treatments while ensuring it is statistically appropriate.

In a two-simplex design the alpha values of the star points have a range of [−sqrt(2)/2; +sqrt(2)/2], instead of [−sqrt(2); +sqrt(2)], as in a conventional CCD. This difference allowed us to set the real enzyme concentrations at zero for the lowest levels of the box part of the design. On the other hand, a conventional CCD would have required “negative” enzyme concentrations for the star points.

The factors took the following values: −1 (0 μL), −α (30 μL), 0 (100 μL), +α (170 μL), and +1 (200 μL) for **P**; and −1 (0 mg), −α (30 mg), 0 (100 mg), +α (170 mg), and +1 (200 mg) for **H**. The experimental design is reported in Table 9.

**Table 9:** Experimental design.

	Coded Value		Uncoded Value	
Run	Protease	Hemicellulase	Protease (μL)	Hemicellulase (mg)
1	−1	−1	0	0
2	1	−1	200	0
3	−1	1	0	200
4	1	1	200	200
5	α	0	170	100
6	−α	0	30	100
7	0	α	100	170
8	0	−α	100	30
9 *	0	0	100	100
10 *	0	0	100	100
11 *	0	0	100	100

* Central point. α alpha value = [sqrt(2)/2].

#### 3.3.2. Statistical Analysis

The data shown in the tables correspond to means and their standard deviations (*n* = 3). The regression and ANOVA models were analyzed according to Montgomery [45]. Pearson’s product-moment correlations between variables were also evaluated. Statistical analyses were performed with R version 3.0.2 (R Core Team, 2013, Vienna, Austria) and Statgraphics Centurion XV (02/15/06 version, 2007, Statpoint Inc, Herndon, VA, Canada) using a critical *p* value of 0.05.

### 3.4. Yield

Yield was calculated as g of product obtained per 100 g of AIR used.

### 3.5. Chemical Analyses

Deionized water was used for all assays. All determinations were performed in triplicate.

#### 3.5.1. Determination of Cellulose, Lignin, Non-Cellulosic Polysaccharides, and Protein in CWM and AIR

Hydrolysis of cellulose and non-cellulosic polysaccharides was performed according to Ng *et al.* [46]. From the supernatants, non-cellulosic carbohydrates and uronic acids were determined. From the residues, lignin and cellulose were determined. Total carbohydrate content was determined by the phenol-sulfuric method [47], and protein content was determined according to Lowry *et al.* [48]. Uronic acid content was determined spectrophotometrically by the method reported by Filisetti-Cozzi and Carpita [49].

#### 3.5.2. Determination of Carbohydrates

The total carbohydrate content was evaluated according to the colorimetric method of Dubois *et al.* [47], using phenol-sulfuric acid and measuring the absorbance at 490 nm. Fructose was used as standard.

#### 3.5.3. Protein Analysis

The protein content in the samples was determined by the assay of Lowry *et al.* [48], using bovine serum albumin (BSA) as standard.

#### 3.5.4. Determination of Uronic Acids

The technique for uronic acids reported by Filisetti-Cozzi and Carpita [49] was used adding sulfamic acid to the samples to suppress the browning of neutral sugars released and heating with a mixture of sulfuric acid and tetraborate.

#### 3.5.5. Determination of Inulin

The determination was performed according to a modification of the method proposed by the AOAC 999.03 and AACC 3232 [50,51]. For this purpose, an enzymatic kit (Fructan Assay Procedure, Megazyme, Ireland) was used to determine fructans.

#### 3.5.6. Determination of Total Polyphenols 

Total polyphenols were assayed using a Folin-Ciocalteau reagent [52]. Results are expressed as gallic acid equivalents (GAE) in g/100g.

#### 3.5.7. Characterization of Polyphenols for Fractions Isolated with Enzymatic Treatment

The isolated fractions (0.4 g) were extracted using methanol (4 mL) and with the help of ultrasound treatment for 45 min at room temperature. Then, the samples were placed on a shaker for 48 h at 25 °C. The extracts were filtered through a 0.45-μm syringe filter.

Polyphenol analysis was conducted with a Waters HPLC consisting of a vacuum degasser, binary pump, column heater, and diode-array detection (DAD) system. The column used was a 150 mm × 4.6 mm i.d., 5 μm particle size, C18 XBridge from Waters (Dublin, Ireland) with a C18 security guard column. Analysis was performed at 40 °C.

The mobile phase was prepared from acidified water (0.5% acetic acid, *v*/*v*) and acetonitrile as eluent A and B. The gradient program was: 0% B (0 min), 20% B (10 min), 30% B (15 min), 50% B (20 min), 75% B (25 min), 100% B (30 min), 0% B (37 min), and finally, the initial conditions were maintained to complete an hour. The flow-rate was 0.8 mL·min^−1^. The injection volume was 20 μL. Monitoring was performed at 320 nm and the diode-array detector was set at an acquisition range from 210 to 800 nm.

For HPLC-MS, separation was performed on an Agilent 1200 Series. This instrument was equipped with a Phenomenex Luna, C18 column (100 × 2.00 mm, 3 μm). The mobile phases and the gradient program were the same as previously described for HPLC-DAD. The flow rate was set at 0.20 mL/min throughout the gradient. The HPLC system was coupled to a Quadrupole-Time-of-Flight mass spectrometer (micrOTOF-Q II, Bruker Daltonik GmbH, Bremen, Germany), an orthogonal accelerated Q-TOF mass spectrometer, equipped with an electrospray ionization source (ESI), which was used in a negative ion mode with nitrogen as the collision gas. The spectra were acquired over a mass ranging from *m*/*z* 50 to 1000. The optimum values of the ESI-MS parameters were: capillary voltage, +3.5 kV; drying gas temperature, 200 °C; drying gas flow, 7.0 L/min; set nebulizer, 51 psi; and collision RF, 150 Vpp.

## 4. Conclusions 

The use of an enzymatic (protease, hemicellulase) treatment performed at 40 °C for 5 h, allowed us to obtain fractions enriched in soluble dietary fiber from the alcohol-insoluble residue of artichoke bracts or stems. The technique is an environmentally friendly method of extraction that contributes to improving the sustainability of the industrialization process.

The yield and chemical composition of fractions were influenced by the tissue used and the level of hemicellulase and protease; the higher levels assayed were the more effective ones for yield or carbohydrate content increases. In general, hemicellulase showed a more marked effect than protease and exerted a positive effect in the carbohydrate content of the fractions.

The application of a heat treatment prior to enzyme action contributed to the increase in yield and inulin content and decreased the protein and polyphenol content of the fractions. In general, interactions between enzymatic and heat treatments were observed for the higher enzyme concentrations used. These fractions, in general, contained the polyphenolic compounds monocaffeoylquinic acid, apigenin, and pinoresinol.

It can be concluded that the isolation procedure proposed can provide fractions enriched in soluble fiber from waste products of artichoke industrialization; the combination of heat and enzymes forms a useful tool for expanding the portfolio of fractions with diverse content of protein, inulin, pectin, and polyphenols, determining different nutritional, biological, and technological functionalities for these fractions.
